# Chemical inhibition of acetyl-CoA carboxylase suppresses self-renewal growth of cancer stem cells

**DOI:** 10.18632/oncotarget.2059

**Published:** 2014-06-05

**Authors:** Bruna Corominas-Faja, Elisabet Cuyàs, Juan Gumuzio, Joaquim Bosch-Barrera, Olatz Leis, Ángel G. Martin, Javier A. Menendez

**Affiliations:** ^1^ Metabolism & Cancer Group, Translational Research Laboratory, Catalan Institute of Oncology, Girona, Catalonia Spain; ^2^ Girona Biomedical Research Institute (IDIBGI), Girona, Catalonia Spain; ^3^ Fundación Inbiomed, San Sebastián, Gipuzkoa Spain; ^4^ Medical Oncology, Catalan Institute of Oncology, Girona, Catalonia Spain; ^5^ StemTek Therapeutics; Bilbao, Biscay Spain

**Keywords:** Acetyl-CoA Carboxylase, Cancer Stem Cells, Lipogenesis, Warburg effect, metabolism, breast cancer, Soraphen A

## Abstract

Cancer stem cells (CSC) may take advantage of the Warburg effect-induced siphoning of metabolic intermediates into *de novo* fatty acid biosynthesis to increase self-renewal growth. We examined the anti-CSC effects of the antifungal polyketide soraphen A, a specific inhibitor of the first committed step of lipid biosynthesis catalyzed by acetyl-CoA carboxylase (ACACA). The mammosphere formation capability of MCF-7 cells was reduced following treatment with soraphen A in a dose-dependent manner. MCF-7 cells engineered to overexpress the oncogene HER2 (MCF-7/HER2 cells) were 5-fold more sensitive than MCF-7 parental cells to soraphen A-induced reductions in mammosphere-forming efficiency. Soraphen A treatment notably decreased aldehyde dehydrogenase (ALDH)-positive CSC-like cells and impeded the HER2's ability to increase the ALDH^+^-stem cell population. The following results confirmed that soraphen A-induced suppression of CSC populations occurred through ACACA-driven lipogenesis: a.) exogenous supplementation with supraphysiological concentrations of oleic acid fully rescued mammosphere formation in the presence of soraphen A and b.) mammosphere cultures of MCF-7 cells with stably silenced expression of the cytosolic isoform ACACA1, which specifically participates in *de novo* lipogenesis, were mostly refractory to soraphen A treatment. Our findings reveal for the first time that ACACA may constitute a previously unrecognized target for novel anti-breast CSC therapies.

## INTRODUCTION

Accumulating evidence indicates that the metabolic state of cancer stem cells (CSCs), a population of cancer cells capable of enhanced self-renewal, governs their tumor-initiating ability. Additionally, CSCs are resistant to conventional therapy and are significantly different from their differentiated cellular counterparts. Because metabolic reprogramming may control the ability of CSCs to avoid treatment and promote recurrence, successful metabolic therapy may eliminate these key drivers of tumor formation, progression, and recurrence [1-6].

Several attempts have been made to therapeutically modulate the metabolic state of cancer cells by treating with compounds that inhibit the most recognizable bioenergetic feature of tumor cells, *i.e.,* aerobic glycolysis (the Warburg effect) [7-11]. However, efforts to inhibit glycolysis using the glucose analog 2-deoxyglucose (2-DG), which accumulates in cells and inhibits glycolytic hexokinase (KH), or the small molecule dichloroacetate (DCA), which inhibits mitochondrial pyruvate dehydrogenase kinase (PDK) and forces pyruvate into the mitochondria to increase mitochondrial metabolism, remain unsatisfactory. In addition, these approaches are not selective for either CSCs or more differentiated bulk tumor cells, and drugs that inhibit glycolysis do not necessarily result in increased mitochondrial metabolism and could result in the disruption of energy production and non-selective cell death. Thus, glycolysis inhibitors may be undesirably toxic to non-cancerous tissues that depend on glycolysis for energy production (*i.e.,* skeletal muscle or brain tissues). CSCs are known to contain lower reactive oxygen species (ROS) levels than their cancerous epithelial-like progeny cells [12]. Therefore, one therapeutic alternative to consider is the re-activation of mitochondrial function and biogenesis, which in turn would impact the suppression of ROS-induced killing in CSCs, as opposed to acutely inducing energy starvation and cell death in all tissues utilizing glycolysis for energy production. In particular, the mitochondrial regulator metformin has been increasingly recognized as a strong therapeutic capable of targeting CSCs in pre-clinical models of human cancer [13-23].

Another possible treatment approach is related to the commonly observed upregulation of endogenous lipid biosynthetic pathways in cancer tissues. This so-called lipogenic phenotype fuels membrane biogenesis in rapidly proliferating cancer cells and renders cancer membrane lipids more saturated. The lipogenic phenotype also impacts fundamental cellular processes associated with cancer cell transformation, including signal transduction, gene expression, ciliogenesis, and response to therapy [24-30]. In the fatty acid synthesis pathway, acetyl-CoA is carboxylated to malonyl-CoA by acetyl-CoA carboxylase (ACACA). Both acetyl-CoA and malonyl-CoA are then used in a condensation reaction by the main lipogenic enzyme fatty acid synthase (FASN) to produce long-chain fatty acids. Of note, it is known that higher expression levels of lipogenic genes and proteins such as FASN are found in CSC subpopulations of breast cancer cell lines and that upregulation of *de novo* fatty acid biogenesis is a pre-requisite for the formation of pre-malignant lesions due to increased CSC survival [31-35]. Moreover, recent studies performed in induced pluripotent stem cells (iPSCs) have revealed that when activities of the ACACA and FASN lipogenic enzymes are inhibited, the efficiency of somatic reprogramming to stemness is decreased [30]. Coincidentally, ACACA and FASN are highly expressed in iPSCs. We recently hypothesized that the stemness features of cancer cells may take advantage of the Warburg effect-related ability of tricarboxylic acid (TCA) cycle intermediates to be siphoned into lipid biosynthesis metabolism for CSC self-renewal and survival.

To test the hypothesis that the therapeutic targeting of endogenous lipogenesis may impact the CSC cellular state in heterogeneous breast cancer cell populations, we examined the polyketide soraphen A, which was chosen for these studies because its mechanism of ACACA inhibition is well defined [36-43]. Unlike RNA interference-based approaches [44], the rapidity of soraphen A-induced inhibition of lipid metabolism minimizes non-specific or adaptive changes caused by changes in cell fatty acid composition and cell growth. Our current results are the first to show that soraphen A treatment can inhibit the formation of mammospheres in a fatty acid-dependent manner, highlighting the potential value of ACACA as a novel metabolic target in breast CSC.

## RESULTS

### Soraphen A decreases mammosphere formation in MCF-7 breast cancer cells

**Figure 1:**
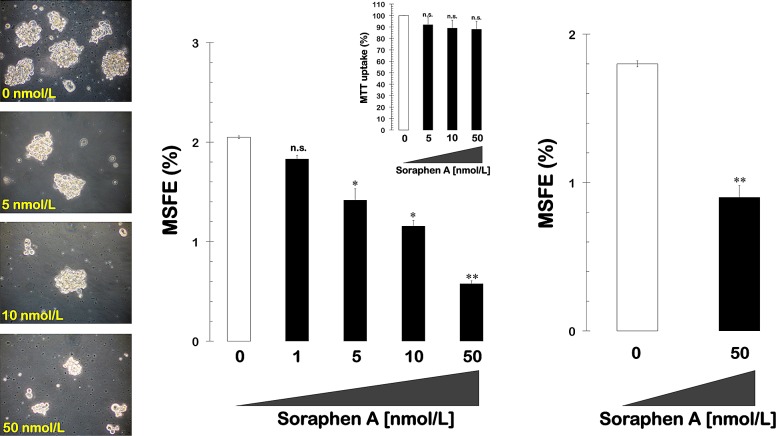
Pharmacological blockade of ACACA activity inhibits mammosphere formation in MCF-7 cells *Left.* Figure shows representative light microscope representations of mammospheres formed by MCF-7 cells growing in sphere medium for 7 days in the absence or presence of graded concentrations of Soraphen A, as specified (20X magnifications). *Middle.* MSFE of MCF-7 cells was calculated as the number of mammospheres (diameter >50 μm) formed in 7 days divided by the original number of cells seeded and expressed as percentage means (*columns*) ± SD (*bars*). Re-feeding of mammospheres cultures with Soraphen A and/or sphere medium was performed on days 3 and 5. The metabolic status of monolayer cultures of MCF-7 cells treated with increasing concentrations of Soraphen A was measured using MTT uptake assays, and cell viability is expressed as % uptake relative to untreated control cells (= 100% cell viability). *Right.* MCF-7 cells were trypsinized into a single cell suspension, and 2 mL was cultured on poly-HEME-coated plates at a density of ~10^5^ cells/mL (total of 2 x 10^5^ cells/well) in the absence or presence of 50 nmol/L soraphen A and then incubated at 37 ^o^C for an additional 48 h. Cells that were visibly anoikis-resistant with intact plasma membranes, *i.e.,* excluding trypan blue stain, were then cultured in soraphen A-free mammosphere medium for 7 days following the same procedure as described above. The results are presented as the mean (*columns*) ± SD (*bars*) of 2 independent experiments performed in triplicate. *P < 0.01 and **P < 0.001, statistically significant differences from the control group. n. s. not statistically significant.

We first tested the ability of MCF-7 breast cancer cells to form tumor spheres when grown in suspension cultures in the presence of a range of concentrations of soraphen A (1, 5, 10, and 50 nmol/L). The MSFE was calculated as the number of sphere-like structures (diameter >50 μm) divided by the original number of cells seeded and expressed as the mean percentage (±SD). A subset (2.0 ± 0.01%) of untreated MCF-7 breast cancer cells formed *bona fide* mammospheres upon initial plating, and this result confirms previous reports that MCF-7 cell cultures intrinsically contain a SC-like population. Interestingly, the spheroid formation capability of MCF-7 cells was significantly reduced following treatment with increasing concentrations of soraphen A in a dose-dependent manner (50% reduction at 15 nmol/L soraphen A; Fig. 1, *left* and *middle panels*). Although the ability of some CSC-like cellular states to survive and proliferate as floating spherical colonies under anchorage-independent conditions at low frequencies (1-3%) is commonly regarded as an *in vitro* surrogate of the self-renewal and tumor-initiating capacity exclusively possessed by CSCs, it should be acknowledged that mammosphere formation assays can also be viewed as *bona fide* assays for evaluating the number of anoikis-resistant cells within heterogenous cancer populations. To unambiguously validate the anti-CSC effects of soraphen A, anoikis was induced by plating MCF-7 cells into culture dishes that had been coated with poly-2-hydroxyethyl methacrylate (Poly-HEME) in the absence or presence of soraphen A (50 nmol/L; 48 h) prior to replating a viable fraction of the suspended samples in serum- and soraphen A-free mammosphere medium. Remarkably, soraphen A-treated anoikis cultures reduced by 50% their mammosphere-formation efficiency after removal of the drug (Fig. 1, *right panel*). Importantly, the soraphen A-induced reduction in mammosphere formation was not due to non-specific toxicity, as MTT-based experiments in monolayer cultures of MCF-7 cells showed that cell viability remained as high as 90% in the presence of identical nanomolar concentrations of soraphen A (Fig. 1, *middle panels*).

### Soraphen A eliminates the HER2-enhanced formation of mammospheres

**Figure 2:**
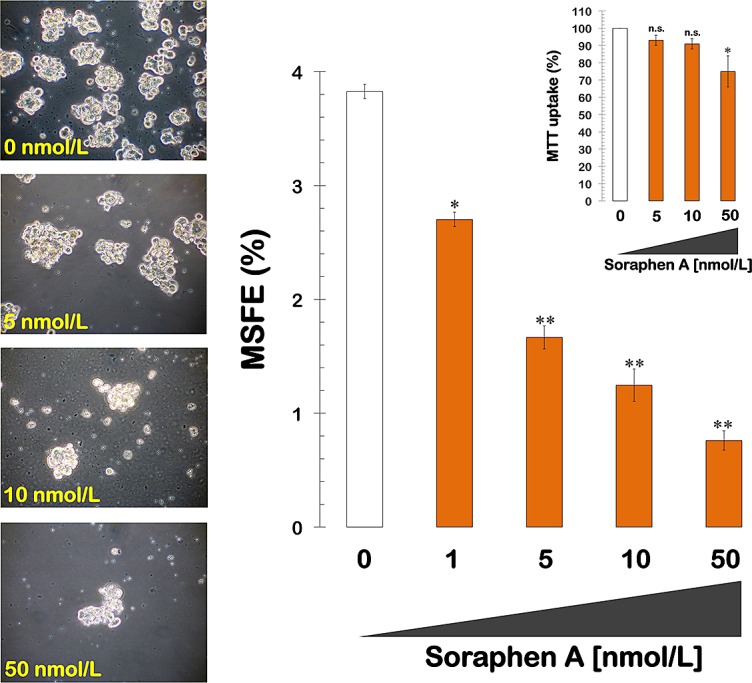
Pharmacological blockade of ACACA activity inhibits mammosphere formation in MCF-7/HER2 cells *Left.* Figure shows representative light microscope representations of mammospheres formed by MCF-7/HER2 cells growing in sphere medium for 7 days in the absence or presence of graded concentrations of Soraphen A, as specified (20X magnifications). *Right.* MSFE of MCF-7/HER2 cells was calculated as the number of mammospheres (diameter >50 μm) formed in 7 days divided by the original number of cells seeded and expressed as percentage means (*columns*) ± SD (*bars*). Re-feeding of mammospheres cultures with Soraphen A and/or sphere medium was performed on days 3 and 5. The metabolic status of monolayer cultures of MCF-/HER2 cells treated with increasing concentrations of Soraphen A was measured using MTT uptake assays, and cell viability is expressed as % uptake relative to untreated control cells (= 100% cell viability). The results are presented as the mean (*column*s) ± SD (*bars*) of 2 independent experiments performed in triplicate. *P < 0.01 and **P < 0.001, statistically significant differences from the control group. n. s. not statistically significant.

We next examined whether treatment with soraphen A was sufficient to prevent the well-recognized ability of the oncogene HER2 to expand the breast CSC population. MCF-7 cells engineered to overexpress HER2 (MCF-7/HER2) exhibited a significantly increased ability to form mammospheres (3.8 ± 0.1%) compared to parental MCF-7 cells. However, this enhanced spheroid formation capability of MCF-7/HER2 cells was drastically reduced following treatment with soraphen A, which decreased the MSFE by 50% at ~3 nmol/L (*i.e.,* a Soraphen A concentration 5 times lower than that needed to decrease MSFE by 50% in MCF-7 parental cells). The highest dose of the soraphen A (50 nmol/L) elicited inhibitory effects of ~80% compared to the basal MSFE found in untreated MCF-7/HER2 tumor sphere cultures (Fig. 2). Mammosphere formation was also drastically decreased by soraphen A treatment in SKBR3 cells, which are a natural model of HER2 overexpression and HER2 dependency for cell proliferation and survival (data not shown).

Similar to the results obtained with MCF-7 cells, the reductions in mammosphere formation were not due to non-specific soraphen A toxicity, as identical concentrations of the drug had no drastic impact on MCF-7/HER2 cell viability under adherent culture conditions. These findings strongly suggest that the mechanism of action targeted by nanomolar concentrations of soraphen A is not essential for the bulk breast cancer cell population.

### Soraphen A decreases the percentage of breast cancer cells expressing the CSC marker ALDEFLUOR

**Figure 3:**
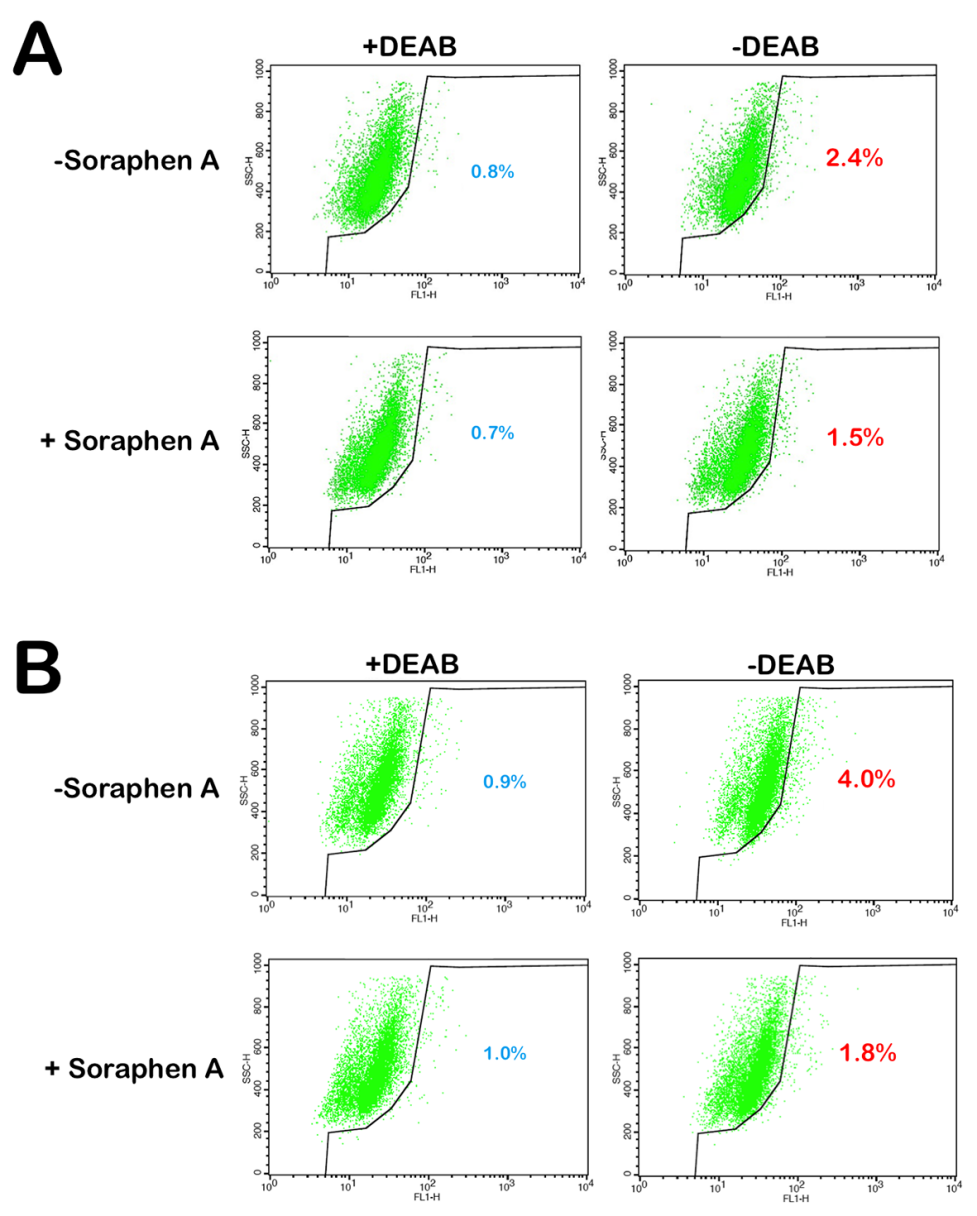
Pharmacological blockade of ACACA activity impedes HER2-induced expansion of stem cell population MCF-7 cells (A) and HER2-overexpressing MCF-7/HER2 cells (B) were subjected to the ALDEFLUOR^®^ assay to identify cells with high ALDH activity in the absence or presence or Soraphen A (10 nmol/L, 3 days with daily re-feeding). The ALDH inhibitor DEAB was used as a negative control. The cells without inhibitor shifted to the right and were considered ALDH-positive cells.

The drastic decrease in mammosphere formation rates observed following treatment of MCF-7 and MCF-7/HER2 cancer cell populations with Soraphen A certainly provided preliminary insight as to the Soraphen A's mechanism of action, in particular to its putative ability to specifically remove CSCs from the bulk population. We therefore envisioned that Soraphen A might specifically suppress aldehyde dehydrogenase (ALDH)-positive CSC-like cells. Using flow cytometry and the ALDEFLUOR^®^ reagent, we first confirmed the presence of an enhanced aldefluor-positive sub-population in MCF-7 and MCF-7/HER2 cells. In Soraphen A-naïve MCF-7 cell populations, approximately 2.4% of the cells expressed high ALDH activity (Fig. 3A). Interestingly, in Soraphen A-treated MCF-7 cell populations, solely 1.5% of the cells remained ALDH-positive. We confirmed that overexpression of HER2 increased the aldefluor-positive population almost twofold compared to MCF-7 parental cells; remarkably, the aldefluor-positive cell content drastically decreased from 4% in Soraphen A-naïve MCF-7/HER2 cell populations to 1.8% in Soraphen A-treated MCF-7/HER2 cells (Fig. 3B).

### Soraphen A suppresses HER2-enhanced mammosphere formation by blocking endogenous lipogenesis

**Figure 4:**
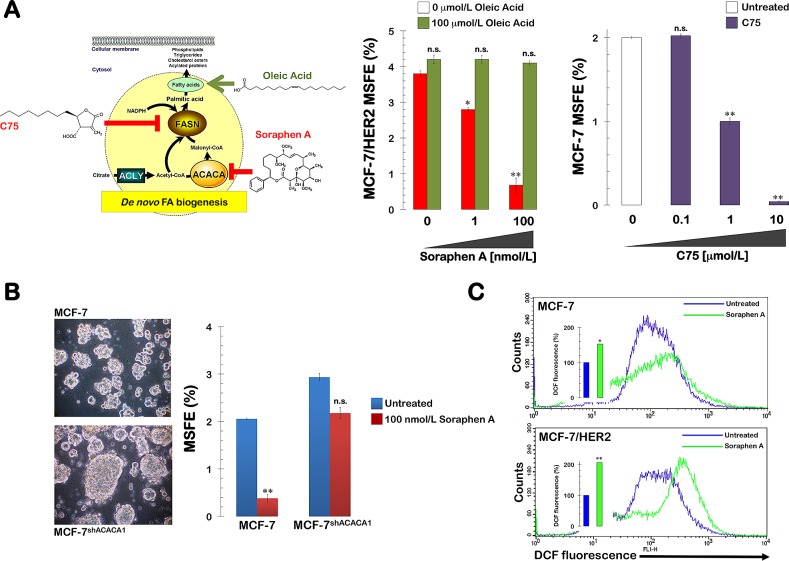
Soraphen A suppresses self-renewal growth of CSC-like cells by blocking ACACA-catalyzed endogenous lipogenesis A. *Right.* MSFE of MCF-7/HER2 cells was calculated as the number of mammospheres (diameter >50 μm) formed in 7 days in the absence or presence of graded concentrations of Soraphen A and co-exposed to 100 μmol/L oleic acid, as specified, divided by the original number of cells seeded and expressed as percentage means (*columns*) ± SD (*bars*). Re-feeding of mammospheres cultures with sphere medium, Soraphen A, and/or oleic acid was performed on days 3 and 5. *Left.* MSFE of MCF-7 cells was calculated as the number B. MSFE of MCF-7 and MCF-7^shACACA1^ cells was calculated as the number of mammospheres (diameter >50 μm) formed in 7 days in the absence or presence of 100 nmol/L Soraphen A divided by the original number of cells seeded and expressed as percentage means (*columns*) ± SD (*bars*). Re-feeding of mammospheres cultures with Soraphen A and/or sphere medium was performed on days 3 and 5. The results in A and B are presented as the mean (*columns*) ± SD (*bars*) of 2 independent experiments performed in triplicate. *P < 0.01 and **P < 0.001, statistically significant differences from the control group. n. s. not statistically significant. C. MCF-7 and MCF-7/HER2 cells, untreated or treated for 3 days with 50 nmol/L soraphen A, were exposed for 60 min to H_2_DCF-DA and their fluorescence intensity was measured by flow cytometry. Figure shows representative scatter plot histograms of DCF fluorescence. The columns at the interior the histograms show relative measurements of ROS levels in soraphen A-treated MCF-7 and MCF-7/HER2 cells compared to untreated controls (=100%). *P < 0.01 and **P < 0.001, statistically significant differences from the control group.

We next sought to unambiguously establish that soraphen A suppresses mammosphere formation by inhibiting *de novo* fatty acid biogenesis (Fig. 4A, *left panel*). Therefore, we determined the rescue potential of oleic acid when the lipogenic pathway was specifically blocked through treatment with soraphen A. The strong inhibitory effects of soraphen A on the mammosphere formation capability of MCF-7/HER2 cells were fully counteracted by supplementation of the mammosphere medium with micromolar concentrations of oleic acid (100 μmol/L; Fig. 4A, *middle panel*).

Notably, in the presence of a gradient of non-cytotoxic micromolar concentrations of the FASN inhibitor C75, MCF-7 cells lost their ability to develop mammospheres in a dose-dependent manner with the highest dose of C75 (10 μmol/L) eliciting consistently greater inhibitory effects than the lowest dose (0.1 μmol/L). Indeed, 10 μmol/L C75 dramatically suppressed the MSFE by > 90% (Fig. 4A, *right panel*).

### Soraphen A suppresses mammosphere formation *via* ACACA

We next evaluated whether soraphen A-induced inhibition of mammosphere formation occurred through the specific targeting of ACACA. To do so, we reevaluated the effects of soraphen A on the mammosphere capability of MCF-7 cells in which the *ACACA* gene was stably silenced via lentiviral-delivered small hairpin RNA (MCF-7^shACACA1^). First, we confirmed differences in mammosphere formation between conditions of ACACA chemical inhibition *versus ACACA* gene silencing. A previous study reported that knockdown of *ACACA* recapitulated AMPK activation and facilitated anchorage-independent growth [45]. Our data indicated that *ACACA* silencing elicited stronger mammosphere formation in MCF-7^shACACA1^ cells (Fig. 4B). In addition, we observed that the enhanced mammosphere formation ability of MCF-7^shACACA1^ cell was mostly refractory to soraphen A treatment even at concentrations of 100 nmol/L, which were able to fully suppress the mammosphere formation capability of MCF-7 parental cells.

## DISCUSSION

Frustrated by the gene-centric limitations of conventional approaches used to identify molecular markers associated with rare tissue-specific sub-populations of CSCs, we recently postulated that CSCs may display unique metabolic features that distinguish them from the bulk of tumor cells. We hypothesized that these metabolic properties may constitute a basis for developing new therapeutic strategies to eliminate CSCs [1]. One of the properties of stem/progenitor cells is their ability to survive under anchorage-independent conditions and generate mammospheres. Our current data are the first to show a highly significant decrease in mammosphere formation in the presence of the polyketide fungicide soraphen A. These results confirm that the lipogenic enzyme ACACA might represent a novel target for anti-breast CSC therapies. These findings, together with the ability of chemical inhibitors of FASN activity such as C75 to recapitulate the anti-mammosphere activity of the ACACA inhibitor soraphen A, also confirm that *de novo* fatty acid biogenesis, a pathway that is frequently deregulated during breast carcinogenesis, plays a functional role in CSC self-renewal and survival.

Therapeutic exploitation of the lipogenic pathway in cancer should consider the effects of blocking one metabolic enzyme on the expression and activity of other metabolic genes. In particular, it is essential to consider that alternate mechanisms/pathways might compensate for the inhibition of any enzyme in the lipogenic pathway. For example, silencing of ATP citrate lyase (ACLY), a cytosolic enzyme that converts citrate into a shared precursor for fatty acid and mevalonate synthesis such as acetyl-CoA, is sufficient to counteract stem cell characteristics induced in diverse cancer cell systems. Moreover, the expression of *FASN,* which catalyzes the rate-limiting reaction for fatty acid synthesis, and *HMGCR,* which is the rate-limiting enzyme in the mevalonate pathway, were both shown to be significantly enhanced after ACLY knockdown. These results suggest that ACLY deficiency forces cancer cells to upregulate the expression of downstream genes in fatty acid and cholesterol synthesis pathways to compensate for the loss of ACLY. Of note, this type of compensation that occurs during the therapeutic targeting of lipogenesis-related enzymes such as ACLY might be unacceptable for cancer treatment [43, 46]. We acknowledge that our study failed to evaluate whether the chemical perturbation of ACACA promoted major changes in the expression profile of other metabolic genes. However, the rapidity of soraphen A-induced inhibition of *de novo* fatty acid biogenesis appeared to minimize the occurrence of non-specific or adaptive changes that could be switched on in response to changes in cell fatty acid composition and cell growth. In fact, the chemical inhibition of ACACA mediated by soraphen A promoted the opposite effect on mammosphere formation as stable genetic silencing of the cytosolic isoform ACACA1, which specifically participates in *de novo* lipogenesis. As reported in the study by Jeon *et al.* [45], knockdown of ACACA was found to recapitulate AMPK activation and facilitate anchorage-independent growth. Thus, MCF-7 cells with stably silenced expression of the cytosolic isoform ACACA1 demonstrate enhanced mammosphere formation capacity. Interestingly, MCF-7^shACACA1^ cells were largely refractory to concentrations of soraphen A that completely suppressed mammosphere formation in MCF-7 parental cells. This finding, as well as the data showing that exogenous supplementation with supraphysiological concentrations of the monounsaturated fatty acid oleic acid fully rescued the ability of MCF-7/HER2 cells to form mammospheres in the presence of soraphen A, mechanistically confirm that soraphen A-induced suppression of breast CSC populations specifically occurs *via* blockade of ACACA1-driven lipogenesis.

Soraphen A-induced inhibition of ACACA has been shown to augment the accumulation of unsaturated fatty acids derived from saturated and polyunsaturated fatty acid precursors by impacting both fatty acid elongation and by disrupting pathways where both elongases and desaturases are utilized. The inhibition of ACACA leads to a significant shift in fatty acid metabolism, whereby saturated fatty acids and essential fatty acids are desaturated but not elongated [42]. Thus, it is tempting to speculate that unlike RNA interference-based approaches [45], the rapid ability of soraphen A to acutely disrupt a strong functional link between ACACA activity and the synthesis of saturated-, monounsaturated-, and polyunsaturated fatty acids through the control of fatty acid elongase activity might underlie soraphen A-mediated elimination of breast CSCs. Other studies have found that reversing the lipogenic switch in cancer cells with soraphen A treatment leads to a marked decrease in saturated and mono-unsaturated phospholipid species and an increase in the relative degree of polyunsaturation. Because polyunsaturated acyl chains are more susceptible to peroxidation, soraphen A-induced acute inhibition of lipogenesis may increase the levels of peroxidation end products and render cells more susceptible to oxidative stress-induced cell death. One of the properties of anoikis-resistant stem/progenitor cells is their ability to survive in anchorage-independent conditions and generate mammospheres. Thus, the soraphen A-induced acute blockade of endogenous lipogenesis may impair the intrinsic ability of CSCs to escape anoikis due to increased oxidative damage and starvation and cell death in detached microenvironmental conditions. In this regard, exposure of breast cancer cells to soraphen A led to a marked augmentation of cells ability to oxidize H_2_DCF-DA (Fig. 4C); because H_2_DCF oxidation by ROS results in formation of strongly fluorescent DCF which is considered to be a marker of ROS abundance and low levels of ROS maintain characteristics of ROS, it might be tempting to suggest that increasing ROS might be part of the pharmacological mechanism through which soraphen A promotes the loss of breast CSCs. Additionally, soraphen A may alter the formation and functioning of membrane microdomains such as lipid rafts, a recently recognized mechanism involved in the escape from anoikis-induced cell death [47-52].

Chemoresistant cell subpopulations from breast cell lines have been shown to possess high levels of ALDH activity. By virtue of those high levels of ALDH activity, FACS can track chemoresistant CSC-like subpopulations as ALDH-positive cells. Indeed, in several types of tumors including breast cancer, cancer cell subpopulations that are enriched for cancer-initiating activity have been readily identified by flow cytometry analysis using the ALDEFLUOR^®^ reagent to identify cells with high levels of ALDH activity [53-56]. We confirmed earlier studies showing that HER2-overexpressing breast cancer cells contain readily detectable higher amounts of ALDH-positive cells [57]. What we believe is remarkable here is that treatment with no cytotoxic concentrations of Soraphen A notably reduced the number of ALDH-positive cells to those levels commonly found in breast cancer cell lines expressing the lowest level of ALDH activity. Moreover, Soraphen A treatment was sufficient to impede the HER2's ability to increase the stem cell population. Therefore, in the presence of Soraphen, the decrease in ALDH activity may account, at least in part, for the loss of stemness of breast CSCs. This is very interesting since the ALDH-positive cells represent the chemosensitive fraction of biologically aggressive breast tumors and it indicates the potential of Soraphen A-like molecules for eliminating ALDH-positive chemoresistant cell subpopulations.

In summary, our findings confirm the importance of *de novo* fatty acid biogenesis in CSC self-renewal and survival and reveal for the first time that ACACA might constitute a previously unrecognized target for novel anti-breast CSC therapies. A large number of nanomolar small-molecule ACACA inhibitors have been developed, and several have been evaluated in clinical trials for metabolic diseases such as obesity and metabolic syndrome [58-60]. The potency and mechanism of ACACA inhibition by soraphen A strongly suggest that pharmacological targeting of the soraphen A binding subunit dimerization site (*i.e.*, the biotin carboxylase (BC) domain of the enzyme) may be useful to identify potent, efficacious ACACA inhibitors for cancer intervention. Using state-of-the art structure-based drug design and crystal structures of human ACACA BC domain, Nimbus Discovery (Cambridge, MA) recently identified a unique series of allosteric inhibitors with low nanomolar potency (ND654, ND646) that bind to the soraphen A binding site. These inhibitors show anti-neoplastic properties against human non-small cell lung cancer (NSCLC) and hepatocarcinoma cells *in vitro* and *in vivo* [61, 62]. If further studies support the anti-CSC activity of these drug-like allosteric inhibitors to bind the BC domain of ACACA with high potency and selectivity, our current findings may open new avenues for exploring potential anti-lipogenesis treatments to successfully target breast CSCs.

## MATERIALS and METHODS

### Drugs and reagents

Soraphen A purified from the myxobacterium *Sorangium cellulosum* was kindly provided by Drs. Klaus Gerth and Rolf Jansen (Hemholtz Zentrum für Infektionsforschung GmbH, Braunschweig, Germany). Oleic acid was purchased from Sigma. C75 was purchased from Alexis Biochemicals (San Diego, CA). Soraphen A and C75 were dissolved in DMSO, and stored in the dark as stock solutions at -20 ^o^C until utilization.

### Culture conditions

MCF-7 human breast cancer cells were obtained from the American Type Culture Collection (ATCC). The cells were routinely grown in improved MEM (IMEM;BioSource International; Invitrogen S.A., Barcelona, Spain) supplemented with 5% fetal bovine serum (FBS) and 2 mmol/L L-Glutamine. The cells were maintained at 37°C in a humidified atmosphere of 95% air and 5% CO_2_. The construction of pBABE/HER2 retroviruses and retroviral infection of MCF-7 cells to generate MCF-7/pBABE and MCF-7/HER2 cells have been described elsewhere [44]. The cells were screened periodically for *Mycoplasma* contamination.

### Mammosphere culture

Mammospheres were generated from single cells of the MCF-7, MCF-7/HER2, and MCF-7^shACACA1^ cell lines seeded at 10^3^ cells/cm^2^ in six-well ultralow attachment plates (Corning Inc.). The sphere medium consisted of F-12/DMEM containing 5 mg/mL insulin, 0.5 mg/mL hydrocortisone, 2% B27 (Invitrogen Ltd.), and 20 ng/mL epidermal growth factor. The medium was made semi-solid by the addition of 0.5% methylcellulose (R&D Systems, Minneapolis, MN) to prevent cell aggregation.

### Mammosphere-forming efficiency

The mammosphere-forming efficiency (MSFE) was calculated as the number of sphere-like structures (large diameter >50 μm) formed in 7 days divided by the original number of cells seeded and expressed as a percentage (mean ± SD).

### Anoikis Assay

6-well tissue plates were coated with 200 μl (12 mg/mL in 95% ethanol) of poly-(2-hydroxyethyl methacrylate) (poly-HEME; Sigma) by incubation overnight at 40 ^o^C. To perform the anoikis assay, MCF-7 cells were trypsinized into a single cell suspension, and 2 mL was cultured in poly-HEME-coated plates at a density of ~10^5^ cells/mL (total of 2 x 10^5^ cells/well) in the absence or presence of 50 nmol/L soraphen A in Dulbecco's Modified Eagle's medium with 10% fetal bovine serum.

### Metabolic status assessment (MTT-based cell viability assays)

Cell viability was determined using a standard colorimetric MTT (3-4,5-dimethylthiazol-2-yl-2, 5-diphenyl-tetrazolium bromide) reduction assay. Exponentially growing cells were harvested by trypsinization, seeded at a concentration of ~2.5 x 10^3^ cells/200 μL/well in 96-well plates, and allowed to attach overnight. The medium was then removed, and fresh medium containing various concentrations of soraphen A was added to the cultures as specified. Control cells without soraphen A were cultured in parallel using the same conditions with comparable media changes. Following treatment (5 days), the medium was removed and replaced with fresh drug-free medium (100 μL/well), and MTT (5 mg/mL in PBS) was added to each well at a 1/10 volume. After incubation for 2–3 h at 37°C, the supernatants were carefully aspirated, and 100 μL of DMSO was added to each well. The plates were agitated to dissolve the crystal product. The optical density (OD) was measured at 570 nm in a multi-well plate reader. The cell viability effects resulting from exposure to soraphen A were analyzed as percentages of the control cell absorbances, which were obtained from control wells treated with appropriate concentrations of the soraphen A vehicle and processed simultaneously. For each treatment, cell viability was evaluated as a percentage using the following equation: (OD_570_ of treated sample/OD_570_ of untreated sample) x 100.

### ALDEFLUOR^®^ activity assay

The ALDEFLUOR^®^ assay (Stem Cell Technologies) quantifies ALDH activity by measuring the conversion of the ALDH substrate BODIPY aminoacetaldehyde to the fluorescent product BODIPY aminoacetate. Briefly, cells were suspended in ALDEFLUOR assay buffer containing the fluorescent ALDH substrate BODIPY-aminoacetaldehyde (BAAA) and incubated for 45 min at 37 ^o^C. The assay buffer also contained a transport inhibitor to prevent efflux of BAAA from the cells. BAAA passively diffuses into live cells and is then converted by intracellular ALDH into a negatively charged product (BODIPY-aminoacetate) that is retained inside cells, labeling the cells with a bright fluorescent signal. After a washing step, the brightly fluorescent ALDH-expressing cells (ALDH^bright^) were detected in the green fluorescence channel (FL1; 520–540 nm) on a FACSCalibur instrument (BD Biosciences). A sample of cells was further stained with a specific ALDH inhibitor, diethylaminobenzaldehyde (DEAB) (Sigma), to serve as a negative control for each experiment. Because only cells with an intact cellular membrane can retain the ALDH1 reaction product, only viable ALDH^bright^ cells were identified. Cells incubated with BAAA and DEAB were used to establish the background signal and define the ALDH^bright^ region. Incubation of cells with the substrate in the absence of DEAB induced a shift in the BAAA fluorescence and defined the ALDH^bright^ population.

### Lentiviral transduction

Pre-packaged lentiviral particles that either encoded a non-targeting shRNA (negative shRNA, sc-108080) or sequences specifically targeting the human *ACCα*(ACACA1) gene (sc-40312-V) were purchased from a commercial provider (Santa Cruz Biotechnology, Inc.). To infect MCF-7 cells with virus, the regular medium was replaced with culture medium containing 5 μg/mL polybrene (Santa Cruz Biotechnology, sc-124220). MCF-7 cells were then exposed to lentiviruses for 48 h. The lentiviral shRNA particles also encode a puromycin resistance gene for transduction selection. After infection, the cells were washed and grown in culture medium containing 10 μg/mL puromycin dihydrochloride (Sigma, P9620) for an additional 72 h. The MCF-7 cells were allowed to proliferate for at least 1 week before any experimental procedures. To monitor the lentiviral transduction efficiency and transgene expression during the experiment we incubated additional subsets of MCF-7 cells with lentiviral particles encoding a green fluorescence protein (GFP) reporter (sc-108084). The transduction efficiency (>90%) was calculated as the ratio of the number of GFP-positive cells to the total number of cells from five random visual fields in three independent culture experiments.

### Reactive Oxygen Species (ROS) Detection

Untreated cells as well as cells treated with soraphen A were incubated 60 min with 10 μmol/L 2’,7’-dihydro-dichlorofluorescein-diacetate (H_2_DCF-DA) (Invitrogen/Molecular Probes) at 37°C. Cellular green fluorescence was then measured by flow cytometry. Following oxidation by ROS and peroxides within cells the non-fluorescent substrate H_2_DCF-DA is converted to the highly fluorescent derivative DCF [63-65]. The cell-permeant non-fluorescent H_2_DCF-DA upon cleavage of the acetate moiety by intercellular esterases and oxidation by ROS is converted to strongly fluorescent DCF and thus reports the ROS abundance.

### Immunoblotting

Testing for the total expression level of ACACA1 was performed by immunoblotting procedures using the ACCα (H-76) sc-30212 rabbit polyclonal antibody (Santa Cruz Biotechnology, Inc.) according to the manufacturer's instructions.

### Statistical analysis

The results are presented as the mean ± SD for at least three repeated individual experiments for each group. The analyses were performed using XLSTAT 2010 (Addinsoft^TM^). A P-value ≤ 0.01 was considered statistically significant.
